# A Cautious Integration With AI in the Clinic: A Standardized-Patient Pilot Study of ChatGPT’s Reliability in Hamilton Depression Rating Scale Scoring

**DOI:** 10.31083/AP51483

**Published:** 2026-08-13

**Authors:** Chun-Hung Chang, Szu-Wei Cheng, Wei-Jen Chen, Chung-Wen Chang, Ting-Hui Liu, Jia-Hau Lee, Sheng-Che Lin, Kuan-Pin Su

**Affiliations:** ^1^Department of Psychiatry, An-Nan Hospital, China Medical University, 709 Tainan, Taiwan; ^2^Department of Nursing, Shu-Zen Junior College of Medicine and Management, 82144 Kaohsiung, Taiwan; ^3^Department of Psychiatry, Chi Mei Medical Center, 710 Tainan, Taiwan; ^4^Mind-Body Interface Research Center (MBI Lab & Care), China Medical University Hospital, 40447 Taichung, Taiwan; ^5^Digital Service for Smart Healthcare, Chia Nan University of Pharmacy & Science, 717301 Tainan, Taiwan; ^6^Office of Research and Development, Asia University, 41354 Taichung, Taiwan; ^7^College of Medicine, China Medical University, 40402 Taichung, Taiwan

**Keywords:** artificial intelligence, large language models, Hamilton Depression Rating Scale, standardized patient

## Abstract

**Background:**

Artificial intelligence (AI) integration offers significant potential to improve mental healthcare, however, the reliability of large language models (LLMs) in performing nuanced clinical tasks remains an important and largely unanswered question. This study aimed to evaluate ChatGPT’s performance in scoring the Hamilton Depression Rating Scale (HAMD-21) compared with expert raters using standardized patients (SPs).

**Methods:**

Three senior mental health experts created and portrayed scenarios for ten SPs representing diverse depressive symptom profiles. Recorded interviews were transcribed and used as input for ChatGPT-4o. HAMD-21 scores generated by ChatGPT were compared with those assigned by expert raters and with predefined script-based reference scores. Inter-rater reliability was assessed using intraclass correlation coefficient (ICC), and differences between raters were evaluated using Steiger’s tests.

**Results:**

ChatGPT and the expert raters achieved good-to-excellent reliability for total HAMD-21 scores (experts: ICC = 0.9921; ChatGPT: ICC = 0.9739). However, expert raters achieved perfect ICCs on 11 individual items, whereas ChatGPT achieved perfect agreement on only 2 items. Steiger’s test demonstrated that experts significantly outperformed ChatGPT on 10 individual items as well as on total scores (Z = 1.931, *p* = 0.0268). Qualitative review revealed that ChatGPT tended to overestimate scores on items related to insomnia and somatic symptoms (items 4–6 and 13) and frequently miscalculated total scores.

**Conclusions:**

ChatGPT demonstrated excellent agreement on total HAMD-21 scores in structured, text-based depression assessments, supporting the potential role of LLMs as adjunctive tools for standardized depression severity evaluation. However, item-level discrepancies and systematic scoring errors indicate that human oversight remains essential for clinically nuanced interpretation.

## Main Points

1. ChatGPT showed excellent total-score agreement in structured text-based HAMD-21 scoring using standardized patient transcripts.

2. Human experts outperformed ChatGPT at the item level, highlighting the challenge of granular symptom interpretation for large language models.

3. ChatGPT demonstrated recurrent overestimation in insomnia and somatic symptom items and occasional total-score calculation errors.

4. Large language models may serve as adjunctive tools for standardized depression severity assessment but require human oversight.

5. This pilot study offers a reproducible framework for future multimodal, multilingual, and benchmarked evaluations of AI-assisted psychiatric rating tasks.

## 1. Introduction

The application of artificial intelligence (AI) in mental health care offers considerable potential to improve both diagnostic accuracy and service accessibility [1,2]. Among these AI innovations, large language models (LLMs), such as ChatGPT, represent a transformative development with the ability to process and generate human-like language. Built on advanced Transformer architectures and trained on extensive textual corpora, ChatGPT demonstrates remarkable capacity for natural language processing, logical reasoning, and conversational analysis [3,4]. Its healthcare applications are rapidly expanding, from medical education and clinical decision support to diagnostic assistance [5,6,7,8,9,10,11,12]. Despite this potential, little is known about ChatGPT’s reliability in structured psychiatric assessments, where subtle distinctions in symptom severity directly influence clinical care. Establishing whether ChatGPT can complement or approximate expert raters is therefore a critical question for both clinical practice and research [13].

Recent literature has also begun to explore the application of LLMs in mental health–specific tasks, including depression screening, symptom extraction, and clinical interaction modeling [10]. While these studies demonstrate promising capabilities, they also report variability in accuracy, sensitivity to prompt design, and limitations in handling nuanced clinical information [14]. Importantly, few studies have evaluated LLM performance using structured psychiatric rating scales, which require precise interpretation of symptom severity and standardized scoring criteria [15,16]. In this context, our study extends the existing literature by focusing on measurement-based assessment, a core component of modern depression care [17]. By using standardized patient scenarios and a validated clinician-rated scale (21-item Hamilton Depression Rating Scale (HAMD-21)) [18], we provide a more granular evaluation of LLM performance at both the item and total score levels. This approach offers new insights into how AI systems perform in clinically relevant, structured assessment tasks beyond diagnostic classification.

Major depressive disorder (MDD) is one of the leading causes of functional disability worldwide and remains a critical public health challenge. Although effective treatments exist, the response rate to initial therapy is only about 50%, and many patients experience side effects, incomplete remission, or discontinuation due to poor tolerability [19,20]. To optimize outcomes, clinical guidelines recommend measurement-based care, in which clinicians routinely assess depressive symptoms, treatment adherence, and adverse effects to guide treatment decisions [21,22]. Among available instruments, the HAMD-21 is one of the most widely used clinician-rated measures [23]. However, its administration requires trained professionals, is time-consuming, and is subject to inter-rater variability [24]. These limitations highlight the urgent need for innovative approaches that can improve the accessibility, consistency, and efficiency of depression assessment.

Although the HAMD-21 is not a diagnostic tool for Major Depressive Disorder as defined by the Diagnostic and Statistical Manual of Mental Disorders, Fifth Edition (DSM-5) [25], many of its items correspond to core DSM-5 symptom domains, including depressed mood, sleep disturbance, psychomotor changes, and somatic symptoms. At the same time, the HAMD-21 includes additional constructs, such as somatic anxiety and gastrointestinal symptoms, which extend beyond DSM-5 diagnostic criteria. In this context, the HAMD-21 is best conceptualized as a measurement-based assessment tool for symptom severity, rather than a categorical diagnostic instrument. This distinction is particularly relevant for the present study, which evaluates the reliability of AI-assisted scoring in structured symptom assessment rather than diagnostic classification.

Standardized patients (SPs) offer a validated and reproducible methodology for benchmarking diagnostic and assessment skills [26]. In psychiatry, SPs provide consistent and clinically realistic presentations of depressive symptoms [27], making them ideal for evaluating rating scale performance across human and AI raters. By leveraging SPs, it becomes possible to systematically assess ChatGPT’s capacity to apply the HAMD-21, while ensuring clinical validity and reproducibility.

The importance of this study lies among the first systematic evaluations of ChatGPT’s accuracy in depression severity assessment using SPs. By comparing ChatGPT with human experts, we identify both the potential of LLMs to support measurement-based care and the pitfalls that necessitate careful supervision. For clinical care, this work demonstrates how ChatGPT could eventually reduce clinician burden, expand access to standardized assessments, and assist settings with limited psychiatric expertise. For research, our findings establish a reproducible framework for testing AI-based tools in mental health, which could accelerate clinical trial assessments and digital phenotyping initiatives. The aim of this study was therefore to evaluate the performance of ChatGPT in standardized depression assessment, benchmarked against human expert raters.

## 2. Materials and Methods

### 2.1 Standardized Patient Simulations and HAMD-21 Assessment Procedure

**Fig. 1.**
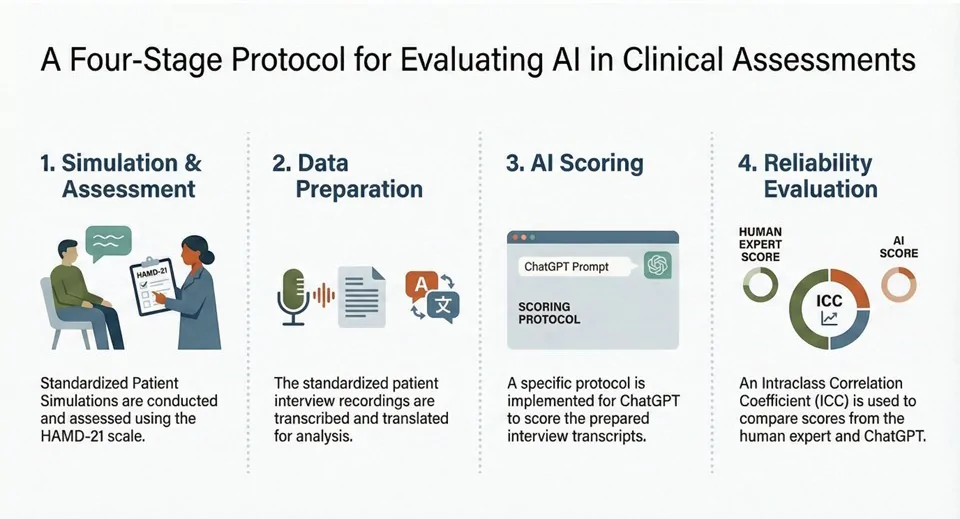
**Four-stage study flowchart for evaluating the reliability of ChatGPT in HAMD-21 assessments**. AI, artificial intelligence.

This study used simulated standardized patient scenarios performed voluntarily by mental health professionals and did not involve real patients, patient medical records, biological specimens, or genetic materials. Three senior psychiatric nurses with extensive clinical and educational experience authored and portrayed the standardized patients (SPs) used in this study. This dual role allowed for the creation of clinically authentic and reproducible SP scenarios. A total of ten standardized patient scripts were developed (Table 1), each depicting a new patient presenting with depressive symptoms of varying severity and clinical characteristics. During each simulation, one expert acted as the standardized patient while another independently served as the evaluator using the HAMD-21. The evaluator did not have access to the predefined script-based HAMD-21 scores during the assessment process. To minimize potential bias, the standardized patient actor and evaluator roles were separated within each simulation session. All evaluators were senior mental health professionals with substantial prior clinical experience in administering and interpreting the HAMD-21 in routine psychiatric practice. All interviews were video-recorded for subsequent transcription and analysis (Fig. 1).

**Table 1.** **Summarizes demographic, clinical, educational information, and HAMD-21 scores for ten standardized patients**.

Case	Sex	Age	Marital status	Education (years)	Simulated HAMD-21 score (script)
1	M	59	married	16	2
2	M	51	married	12	5
3	F	25	single	18	27
4	M	28	single	18	5
5	F	25	single	12	9
6	F	60	married	9	9
7	F	49	married	16	26
8	F	42	married	12	17
9	F	43	single	12	21
10	M	29	single	16	22

HAMD-21, 21-item Hamilton Depression Rating Scale; F, female; M, male.

### 2.2 Transcription and Translation of Standardized Patient Interviews for Analysis

Because GPT-4o is limited to text input and cannot process video data, the recorded standardized patient (SP) interviews were first converted into textual form. Each interview underwent a structured, two-step transcription process. First, audio was extracted from the video recordings and transcribed verbatim in Chinese using OpenAI’s Whisper automatic speech recognition (ASR) system (OpenAI, San Francisco, CA, USA; https://openai.com/index/whisper/), implemented via the Google Colab platform (Google LLC, Mountain View, CA, USA). The output was carefully reviewed and formatted to preserve speaker turns, pauses, and clinically relevant verbal cues (e.g., emotional tone or hesitation) to ensure fidelity to the original dialogue. These transcription features were retained to preserve clinically meaningful conversational context for text-based analysis. These finalized transcripts served as the textual input for ChatGPT, GPT-4o version (OpenAI, San Francisco, CA, USA; accessed in November 2024, https://chat.openai.com/) analysis. The ChatGPT prompt structure and example inputs used for the scoring tasks are described in detail in the **Supplementary Material** to facilitate reproducibility.

### 2.3 ChatGPT Scoring Protocol Implementation

Standardized prompts and transcript formatting procedures were consistently applied across all ChatGPT evaluation sessions to improve reproducibility. The HAMD-21 Chinese Manual served as the standardized reference for assessment criteria. Each of the ten transcripts underwent three independent scoring sessions through ChatGPT, with each evaluation conducted in separate chat sessions to reduce carryover effects between evaluations. This pilot study was designed as an exploratory evaluation of ChatGPT reliability in structured HAMD-21 assessments using standardized patient scenarios. Averaged ChatGPT scores were used to reduce stochastic variability across repeated evaluations.

### 2.4 Statistical Analysis

Assessment reliability was evaluated using a two-way random-effects, single-rater, absolute-agreement intraclass correlation coefficient model (ICC(2,1)) between the standardized patient scores and each rater’s assessment (the human experts and the averaged ChatGPT scores), calculated with MedCalc, version 22.023 (MedCalc Software Ltd., Ostend, Belgium). ICCs were transformed using Fisher’s Z transformation and compared using one-tailed Steiger’s test for two dependent correlations [28]. Exploratory one-tailed Steiger’s Z tests were conducted to provide preliminary comparisons of dependent agreement estimates between human raters and ChatGPT. One-tailed tests were selected based on the a priori hypothesis that human expert raters would demonstrate equal or superior reliability compared with ChatGPT. A *p*-value of less than 0.05 was considered statistically significant.

## 3. Results

### 3.1 Demographic and Clinical Characteristics of the Study Population

The study sample comprised ten standardized patients with diverse demographic and clinical presentations. The cohort included four males and six females, with ages ranging from 25 to 60 years (mean age 41.1 years). Half of the participants were married, while the other half were single. Educational attainment varied from 9 to 18 years of formal education, with a median of 14 years. The simulated HAMD-21 scores demonstrated considerable clinical heterogeneity, ranging from minimal depression (score of 2) to severe depression (score of 27), with a mean score of 14.3. There are three mild cases with scores below 8, three moderate cases with scores ranging from 9 to 17, and four severe cases with scores of 18 or higher (Table 1).

### 3.2 The Reliability of Human Expert Evaluations

Individual item and total score ICCs between the human experts and reference script-based scores of standard patients are summarized in Table 2. The human experts achieved a perfect ICC of 1 (indicating perfect agreement) in 11 items. ICCs for the remaining items are mostly above 0.75 (the threshold for good agreement [29]). The ICC for total HAMD-21 scores was 0.9921 (95% confidence interval [CI]: 0.9698 to 0.9980).

**Table 2.** **Comparison of the reliability of human and ChatGPT evaluations**.

HAMD-21	SP vs Human		SP vs ChatGPT		Steiger’s Z	One-tailed *p*-value
	ICC	95% CI	ICC	95% CI		
1	1.0000		0.9362	0.7751 to 0.9836	NA	<0.0001
2	1.0000		0.9370	0.7736 to 0.9839	NA	<0.0001
3	1.0000		0.9446	0.8033 to 0.9858	NA	<0.0001
4	1.0000		0.9865	0.9368 to 0.9968	NA	<0.0001
5	0.8989	0.6649 to 0.9735	0.8571	0.4957 to 0.9633	0.555	0.2894
6	0.8989	0.6649 to 0.9735	0.7493	0.2768 to 0.9309	1.466	0.0713
7	0.8889	0.6268 to 0.9710	0.7455	0.2880 to 0.9291	1.144	0.1263
8	0.6400	0.0917 to 0.8951	0.8421	0.5105 to 0.9577	–1.140	0.1271
9	1.0000		1.0000		0	0.5000
10	0.9684	0.8857 to 0.9919	0.9609	0.8352 to 0.9904	0.278	0.3904
11	0.9379	0.7751 to 0.9841	0.9358	0.7516 to 0.9839	0.043	0.4830
12	1.0000		0.9256	0.7278 to 0.9811	NA	<0.0001
13	0.9396	0.7894 to 0.9844	0.9622	0.8630 to 0.9903	–0.612	0.2702
14	1.0000		0.8696	0.5655 to 0.9658	NA	<0.0001
15	1.0000		0.7716	0.3435 to 0.9370	NA	<0.0001
16	1.0000		0.0000	–0.1432 to 0.3353	NA	<0.0001
17	<0.0001	–0.6021 to 0.6021	0.0000	–0.5089 to 0.5759	0	0.5000
18	0.8615	0.5611 to 0.9632	0.9032	0.6625 to 0.9750	–1.110	0.1336
19	0.7955	0.3966 to 0.9441	0.7492	0.3025 to 0.9299	1.843	0.0327
20	1.0000		0.6875	0.1899 to 0.9101	NA	<0.0001
21	1.0000		1.0000		0	0.5000
Total	0.9921	0.9698 to 0.9980	0.9739	0.8983 to 0.9934	1.931	0.0268

Abbreviations: CI, Confidence Interval; ICC, Intraclass correlation coefficient; SP, Standardized Patient; NA, not applicable. ICC values reported as 0.0000 indicate values very close to zero after rounding.

### 3.3 The Reliability of ChatGPT Evaluations

Individual item and total score ICCs between ChatGPT and standard patients are summarized in Table 2. ChatGPT achieved a perfect ICC of 1 in 2 items. ICCs for the remaining items are mostly above 0.75. The ICC for total HAMD-21 scores was 0.9739 (95% CI = 0.8983 to 0.9934).

### 3.4 Comparing the Reliability of ChatGPT to Human Experts

The results of Steiger’s test are presented in Table 2. The human experts achieved statistically significantly better scores in 10 items, while no significant differences were observed in the remaining items. The human experts also demonstrated a more reliable total HAMD-21 score evaluation than ChatGPT (Z = 1.931, *p *= 0.0268).

### 3.5 Evaluation Discrepancies in ChatGPT HAMD-21 Assessments: Overestimated Score Errors and Total Score Calculation Errors

Table 3 summarizes ChatGPT assessment errors. Overestimated score errors frequently occur in questions 4, 5, 6, and 13 of the HAMD-21 scale, where the severity ranges from 0 to 2 points but is incorrectly rated as 3 points. Additionally, ChatGPT exhibits total score calculation errors when calculating the total HAMD-21 score. The differences between the original script and the professional scores are within 2 points, while the differences between the original script and ChatGPT scores are within 6 points.

**Table 3.** **Summary of ChatGPT’s overestimated score errors and total score calculation errors**.

Evaluation	Overestimated score error (times)	Total score calculation error (times)
First ChatGPT evaluation	1	6
Second ChatGPT evaluation	1	4
Third ChatGPT evaluation	3	0

## 4. Discussion

To our knowledge, this is among the first pilot studies to evaluate ChatGPT performance in HAMD-21 scoring using standardized patient transcripts. The present findings show that ChatGPT achieved excellent total-score agreement with predefined reference scores, suggesting meaningful potential for LLM-assisted structured depression severity assessment in measurement-based care contexts. At the same time, item-level discrepancies and recurrent scoring errors highlight important areas for refinement and the continued need for human oversight.

Recent reviews of ChatGPT and other large language models (LLMs) in clinical medicine highlight both their promise and their limitations. A systematic review of 128 healthcare-related studies reported that, although ChatGPT was generally viewed favorably, its accuracy varied substantially across different medical tasks and specialties [30]. For example, in diagnostic challenge settings, ChatGPT achieved fair to good agreement (Cohen’s κ ≈ 0.63) when identifying final diagnoses from differential lists [31]. Another meta-analysis found that ChatGPT demonstrated an overall pooled accuracy of 56% (95% CI: 51%–60%, I^2^ = 87%) when responding to medical queries, although included studies differed widely in question sources, phrasing, and evaluation metrics [32]. The present study extends this literature by evaluating ChatGPT not in discrete question-answering or diagnostic contexts, but in structured psychiatric severity assessment using the HAMD-21 within standardized patient interviews. The high total-score intraclass correlation coefficient (ICC = 0.9739 for ChatGPT) indicates a level of reliability comparable to that reported in broader medical-LLM applications. The high ICC observed for the HAMD-21 total score should be interpreted cautiously, as aggregation across multiple items may artificially increase overall agreement despite clinically meaningful discrepancies at the individual item level. Although recent studies suggest promising applications of LLMs in mental health assessment and psychiatric decision-making, relatively few investigations have focused on structured clinician-rated symptom scales. Compared with general diagnostic or question-answering tasks, structured depression rating requires precise interpretation of symptom severity and consistent application of scoring criteria. Our study therefore extends the current literature by examining LLM reliability within a measurement-based care framework using the HAMD-21.

ChatGPT demonstrated lower item-level reliability than human experts despite relatively high total-score agreement. At the item level, ChatGPT rarely produced fully accurate scores (2 in 21 items), whereas the human experts achieved perfect agreement in more than half of the items (11 in 21 items; χ^2^ = 9.0239, *p *= 0.0076). These findings suggest that item-level analyses may provide a more sensitive evaluation of AI-assisted psychiatric rating performance than total-score agreement alone. The seemingly good agreement between ChatGPT and standard patient scores is accompanied by wide confidence intervals, likely attributable to the small sample size in our study. Notably, ChatGPT showed near-zero ICCs in item 16 (Loss of weight) and item 17 (Insight), while the human experts demonstrated an ICC of 1 for item 16 and a similarly low ICC for item 17. A close inspection of item 16 revealed that, despite the variability in correct scores, ChatGPT assigned a score of 0 to all standard patients, demonstrating a profound limitation to detect subtle cues related to weight loss from the diagnostic interview transcripts. In contrast, the low ICC for item 17 appears to reflect limited between-subject variance, as most scores were 0. Although ChatGPT demonstrated higher ICCs in item 8, item 13, and item 18, these differences were not statistically significant. At the total score level, the human experts outperformed ChatGPT statistically, achieving a near-perfect ICC (0.9921). Although ChatGPT also achieved an excellent agreement (0.9739) with the standard patient total scores, the Spearman-Brown formula indicates that aggregating multiple item scores tends to inflate reliability estimates [33]. Therefore, these results should be interpreted with caution. The relatively small sample size may have contributed to instability in several item-level ICC estimates, particularly for HAMD-21 items with restricted score distributions or limited between-subject variability. Accordingly, the present findings should be interpreted as preliminary and hypothesis-generating rather than definitive estimates of AI reliability in depression assessment.

The identified scoring inaccuracies reveal important limitations in ChatGPT’s assessment abilities. The consistent overestimation pattern in items 4–6 (insomnia cluster) and item 13 (somatic symptoms), where conditions warranting mild to moderate scores (0–2 points) were erroneously assigned maximum severity (3 points), suggests potential limitations in ChatGPT’s interpretation of severity thresholds. Calculation errors have been reported in previous ChatGPT studies [34,35]. The computational inaccuracies in total score calculations further indicate a systematic limitation in the AI’s mathematical processing capabilities. These patterns likely stem from the underlying training methodology of large language models, which may prioritize pattern recognition over precise numerical operations. In addition, these computational discrepancies may reflect the probabilistic and stochastic nature of large language models, where outputs are generated through next-token prediction rather than deterministic rule-based computation. As a result, numerical calculations and severity threshold calibration may vary across sessions despite similar prompts. Prompt phrasing, transcript formatting, and lack of structured output constraints may also contribute to variability in scoring behavior. Future studies may improve reproducibility by implementing deterministic scoring frameworks, structured response templates, or external arithmetic verification systems.

An additional methodological consideration relates to the transformation of video-based interviews into text transcripts. Human expert raters had access to nonverbal and paralinguistic information during the original interviews, including facial expressions, speech latency, emotional tone, psychomotor behavior, and affective presentation, whereas ChatGPT assessments were limited to text-only input. The absence of these clinically relevant cues may have reduced ecological validity and systematically disadvantaged ChatGPT in certain HAMD-21 items requiring nuanced interpretation of affective or behavioral symptoms. Although the transcription process preserved pauses, hesitation markers, and clinically relevant verbal expressions whenever possible, text-based representations cannot fully capture the richness of real-world psychiatric interviews.

The development of computer-assisted tools over time illustrates both the progress made and the challenges that remain. Early systems relied on rule-based algorithms with limited computational power, struggling to interpret complex linguistic and contextual nuances. They were constrained by static, pre-programmed questionnaires, basic keyword matching, and restricted access to diverse datasets, leading to poor adaptability, accuracy, and generalizability [36]. In contrast, modern AI tools like ChatGPT leverage advanced natural language processing (NLP) and large language models, trained on extensive and diverse datasets. This enables contextual understanding, dynamic interactions, and the ability to probe deeper into nuanced patient responses. These advancements highlight the evolution from rigid, static systems to sophisticated, adaptive tools capable of addressing the complexities of depression assessment. Despite these significant advancements over the past 20–30 years, our study still revealed specific limitations that underscore the continued importance of human oversight in computer-assisted depression assessments. Future AI model refinement may lead to specialized, reliable systems capable of performing more frequent evaluations between routine clinical interviews for psychiatrists to review.

### 4.1 Strengths

A key strength of this study lies in its use of standardized patients (SPs) created and portrayed by experienced mental health professionals, ensuring both clinical realism and methodological reproducibility. This design allowed for precise control over symptom presentation while maintaining the natural variability of real clinical encounters. The verbatim transcription and structured input format further enhanced reproducibility by standardizing the data provided to ChatGPT across cases. Collectively, these methodological features enabled a rigorous and clinically relevant assessment of ChatGPT’s reliability in structured psychiatric rating—offering a reproducible framework for future research on AI-assisted mental health evaluation. Our findings should therefore be interpreted within the framework of symptom severity assessment rather than DSM-5 diagnostic classification, highlighting the importance of evaluating AI performance in measurement-based care tasks.

### 4.2 Future Research Directions

Future studies should validate AI-assisted psychiatric assessment using larger and more diverse clinical populations, independent blinded raters, and real-world psychiatric interviews across multiple healthcare settings. More naturalistic interviews involving comorbid psychiatric conditions, ambiguous symptom narratives, and variable communication styles may help better characterize real-world AI performance and ecological validity. The development of multimodal AI systems capable of integrating audiovisual, behavioral, and linguistic information may further improve the assessment of affective expression, psychomotor symptoms, and other clinically nuanced psychiatric features. In addition, future research should investigate structured reasoning workflows, deterministic response constraints, external arithmetic verification systems, and advanced statistical approaches such as multilevel or mixed-effects modeling to improve reproducibility and better account for repeated-measures dependency structures in AI-generated ratings. Comparative benchmarking studies are also needed to evaluate the relative utility of large language models against rule-based systems, conventional natural language processing methods, alternative machine learning approaches, and multilingual or language-specialized AI models, including Chinese-focused LLMs. Finally, prospective studies integrating AI-assisted scoring into measurement-based care workflows may help clarify the clinical utility, implementation feasibility, and safety considerations of large language models in psychiatric practice.

### 4.3 Limitations

Several limitations should be considered when interpreting the present findings. First, this pilot study included a relatively small number of standardized patient (SP) cases (n = 10), which limits statistical power, may reduce the stability of item-level ICC estimates, and restricts generalizability. Some HAMD-21 items also showed limited score variability, and total HAMD-21 scores, as aggregated measures, may obscure clinically meaningful item-level discrepancies. Therefore, the present findings should be interpreted as preliminary and hypothesis-generating rather than definitive estimates of AI reliability. Second, although expert-authored and portrayed SPs improved methodological standardization and reproducibility, this design may not fully capture the heterogeneity and spontaneity of real-world psychiatric interviews. The structured symptom presentations may have simplified the scoring task, and partial overlap between SP development and expert evaluation roles may have introduced circularity, although evaluators did not have access to the predefined script-based reference scores during assessment. Third, ChatGPT evaluations were based on Chinese-language text transcripts rather than audiovisual interviews. As a result, nonverbal and paralinguistic information, such as affective expression, speech prosody, psychomotor behavior, facial expression, and interpersonal interaction, was unavailable to the model. This may affect ecological validity, particularly for symptom domains that rely partly on nonverbal clinical cues, and may limit generalizability across languages, cultures, healthcare settings, and other multilingual or Chinese-focused language models. Finally, several statistical and AI-methodological issues should be noted. Multiple item-level comparisons were exploratory and were not corrected for multiplicity. Steiger’s Z tests were used as preliminary comparative measures despite the complexity of ICC-based dependent correlation structures. Repeated ChatGPT evaluations were averaged to reduce stochastic variability, but this approach did not fully model hierarchical dependency and may underestimate uncertainty. In addition, only one standardized prompting strategy was evaluated, and no rule-based, conventional NLP, or alternative machine learning baseline was included. Future studies should address these issues using larger real-world datasets, independent raters, multimodal inputs, multilevel modeling, prompt engineering comparisons, and computational benchmarking.

## 5. Conclusions

ChatGPT demonstrated excellent total-score agreement in structured text-based HAMD-21 scoring using standardized patient transcripts, supporting the potential role of large language models as adjunctive tools for standardized depression severity assessment. However, item-level discrepancies, systematic overestimation patterns, and calculation errors indicate that human oversight remains essential, particularly for clinically nuanced symptom interpretation. This pilot study provides an early reproducible framework for evaluating AI-assisted psychiatric rating tasks. By identifying both the promise and the current boundaries of LLM-based scoring, our findings offer a constructive foundation for future large-scale, multimodal, multilingual, and benchmarked studies aimed at advancing AI-supported measurement-based care in psychiatry.

## Data Availability

All data generated or analyzed during this study have been included in the manuscript.
