# Supplementary material for: A Cautious Integration With AI in the Clinic: A Standardized-Patient Pilot Study of ChatGPT’s Reliability in Hamilton Depression Rating Scale Scoring
Source: Alpha Psychiatry. 2026 Aug 13;27(4):51483. doi: 10.31083/AP51483 (PMC13539987; doi:10.31083/AP51483)
Supplement: Supplementary file 1 [file 2757-8038-27-4-51483-s1.zip › Supplementary Material/Supplementary Table 2.docx]

**Supplementary Table 2.** The ICCs between the human expert and ChatGPT scores

| HAMD | ICC | 95% CI |
| --- | --- | --- |
| 1 | 0.9362 | 0.7751 to 0.9836 |
| 2 | 0.937 | 0.7736 to 0.9839 |
| 3 | 0.9446 | 0.8033 to 0.9858 |
| 4 | 0.9865 | 0.9368 to 0.9968 |
| 5 | 0.8907 | 0.6413 to 0.9712 |
| 6 | 0.8343 | 0.4894 to 0.9555 |
| 7 | 0.7519 | 0.2725 to 0.9321 |
| 8 | 0.6538 | 0.1366 to 0.8986 |
| 9 | 1 |  |
| 10 | 0.9335 | 0.6908 to 0.9841 |
| 11 | 0.8579 | 0.4323 to 0.9649 |
| 12 | 0.9256 | 0.7278 to 0.9811 |
| 13 | 0.9109 | 0.6820 to 0.9771 |
| 14 | 0.8696 | 0.5655 to 0.9658 |
| 15 | 0.7716 | 0.3435 to 0.9370 |
| 16 | 0.0000 | -0.1432 to 0.3353 |
| 17 | 0.5000 | -0.1647 to 0.8484 |
| 18 | 0.9735 | 0.8980 to 0.9933 |
| 19 | 0.9929 | 0.9735 to 0.9982 |
| 20 | 0.6875 | 0.1899 to 0.9101 |
| 21 | 1 |  |
| Total | 0.9826 | 0.9354 to 0.9956 |
