# Supplementary material for: A Cautious Integration With AI in the Clinic: A Standardized-Patient Pilot Study of ChatGPT’s Reliability in Hamilton Depression Rating Scale Scoring
Source: Alpha Psychiatry. 2026 Aug 13;27(4):51483. doi: 10.31083/AP51483 (PMC13539987; doi:10.31083/AP51483)
Supplement: Supplementary file 1 [file 2757-8038-27-4-51483-s1.zip › Supplementary Material/Supplementary Text 1.docx]

# Supplementary Materials

## Supplementary Method 1. ChatGPT Prompting and Scoring Procedure

To improve reproducibility and reduce variability across sessions, standardized prompting procedures were used for all ChatGPT evaluations. Verbatim interview transcripts were provided as text input to ChatGPT-4o without additional clinical interpretation or manual preprocessing beyond transcript formatting.

The model was instructed to score each HAMD-21 item individually according to standard HAMD-21 criteria and to provide both item-level scores and a total score interpretation. All evaluations used the same prompt structure and transcript formatting conventions across cases.

Each transcript was independently evaluated three times in separate ChatGPT sessions to reduce stochastic variability. Averaged scores were used for reliability analyses.

Model: ChatGPT-4o (OpenAI). Default generation settings provided by the platform were used.

## Standardized Prompt Template

You are a senior psychiatrist experienced in the Hamilton Depression Rating Scale (HAMD-21).

Please evaluate the following patient interview transcript using the HAMD-21 scale.

For each HAMD-21 item:
1. Assign a score
2. Briefly explain the reason for the score based on the transcript
3. Provide the final total HAMD-21 score
4. Interpret the depression severity

Please follow standard HAMD-21 scoring principles and base your evaluation only on the information explicitly available in the transcript.

## Representative Example of ChatGPT HAMD-21 Scoring Output

Depressed Mood: Mr. Li reported no sadness or desire to cry.
Score: 0

Middle Insomnia: He wakes once or twice nightly to use the bathroom but returns to sleep easily.
Score: 1

Retardation (Psychomotor): He reported mildly slowed thinking.
Score: 1

Total HAMD-21 Score: 2
Interpretation: Minimal depressive symptoms.

Minor calculation inconsistencies occasionally occurred across sessions, including discrepancies between item-level scores and reported total scores.
